# ChatGPT is an Unreliable Source of Peer-Reviewed Information for Common Total Knee and Hip Arthroplasty Patient Questions

**DOI:** 10.1155/aort/5534704

**Published:** 2025-01-06

**Authors:** Jonathan D. Schwartzman, M. Kareem Shaath, Matthew S. Kerr, Cody C. Green, George J. Haidukewych

**Affiliations:** ^1^College of Medicine, University of Central Florida, Orlando, Florida, USA; ^2^Orlando Health Jewett Orthopedic Institute, Orlando, Florida, USA; ^3^Orthopaedic Surgery Department, Cleveland Clinic Florida, Weston, Florida, USA

## Abstract

**Background:** Advances in artificial intelligence (AI), machine learning, and publicly accessible language model tools such as ChatGPT-3.5 continue to shape the landscape of modern medicine and patient education. ChatGPT's open access (OA), instant, human-sounding interface capable of carrying discussion on myriad topics makes it a potentially useful resource for patients seeking medical advice. As it pertains to orthopedic surgery, ChatGPT may become a source to answer common preoperative questions regarding total knee arthroplasty (TKA) and total hip arthroplasty (THA). Since ChatGPT can utilize the peer-reviewed literature to source its responses, this study seeks to characterize the validity of its responses to common TKA and THA questions and characterize the peer-reviewed literature that it uses to formulate its responses.

**Methods:** Preoperative TKA and THA questions were formulated by fellowship-trained adult reconstruction surgeons based on common questions posed by patients in the clinical setting. Questions were inputted into ChatGPT with the initial request of using solely the peer-reviewed literature to generate its responses. The validity of each response was rated on a Likert scale by the fellowship-trained surgeons, and the sources utilized were characterized in terms of accuracy of comparison to existing publications, publication date, study design, level of evidence, journal of publication, journal impact factor based on the clarivate analytics factor tool, journal OA status, and whether the journal is based in the United States.

**Results:** A total of 109 sources were cited by ChatGPT in its answers to 17 questions regarding TKA procedures and 16 THA procedures. Thirty-nine sources (36%) were deemed accurate or able to be directly traced to an existing publication. Of these, seven (18%) were identified as duplicates, yielding a total of 32 unique sources that were identified as accurate and further characterized. The most common characteristics of these sources included dates of publication between 2011 and 2015 (10), publication in The Journal of Bone and Joint Surgery (13), journal impact factors between 5.1 and 10.0 (17), internationally based journals (17), and journals that are not OA (28). The most common study designs were retrospective cohort studies and case series (seven each). The level of evidence was broadly distributed between Levels I, III, and IV (seven each). The averages for the Likert scales for medical accuracy and completeness were 4.4/6 and 1.92/3, respectively.

**Conclusions:** Investigation into ChatGPT's response quality and use of peer-reviewed sources when prompted with archetypal pre-TKA and pre-THA questions found ChatGPT to provide mostly reliable responses based on fellowship-trained orthopedic surgeon review of 4.4/6 for accuracy and 1.92/3 for completeness despite a 64.22% rate of citing inaccurate references. This study suggests that until ChatGPT is proven to be a reliable source of valid information and references, patients must exercise extreme caution in directing their pre-TKA and THA questions to this medium.

## 1. Introduction

Artificial intelligence (AI), machine learning, and large language models (LLMs) have exponentially risen in popularity, research, and public conversation over the past decade. Recent years have seen technological advancements in these spaces become increasingly accessible for companies, consumers, and, notably, patients in the healthcare system [1, 2]. One LLM, ChatGPT-3.5, by OpenAI (San Francisco, California, the United States of America), is an AI chatbot that can provide nuanced dialog on many topics of conversation with a natural, human-sounding interface [3]. ChatGPT can act as a source of information on almost any topic while providing references. Without being specifically prompted to utilize the peer-reviewed literature, ChatGPT will default to utilizing publicly available websites which vary greatly in reliability [4]. With over one million registered users within its first five days of launch and 100 million within the next two months, it is of timely significance that investigations into ChatGPT's methods and output reliability are investigated [5, 6].

OpenAI's open access (OA) version of the chatbot, ChatGPT-3.5, can be utilized by any individual who provides an email address to make an account, prompting many in the medical community to speculate whether patients might direct their questions here rather than or in conjunction with advice provided by healthcare professionals [6]. Recent studies have found that over 89% of citizens in the United States turn to Internet queries before consulting a medical professional [4]. ChatGPT may emerge as a contender in this space because, unlike search engines, which typically provide links to innumerable potential answers, ChatGPT can coalesce several sources of information into a succinct response. This raises the question of ChatGPT's reliability in terms of content and source material. For example, one recent study comparing responses to common questions regarding the total joint arthroplasty procedure between Google Chrome and ChatGPT found “very dissimilar” answers to discrete and open-ended questions [4]. ChatGPT, as opposed to other AI tools such as Perplexity, was used in this study given that it is the most popular LLM used today and is most likely to be used by patients.

The medical community has followed in suit of ChatGPT's exponential rise in popularity and public attention with studies investigating the extent of its medical knowledge. When prompted to respond to a series of orthopedic in-training examination questions meant to replicate the exam, ChatGPT performed at a comparable level to first-year orthopedic surgery residents [7]. On a more generalizable scale in medicine, ChatGPT was found to meet the threshold for a passing score on the United States Medical Licensing Examination Step 1, Step 2CK, and Step 3 [8, 9]. These findings indicate that ChatGPT can utilize at least a passing basis of medical knowledge, but exactly where that information is sourced from remains unknown for any given question or input. Given the significance of peer-reviewed evidence in the medical community, whether ChatGPT is capable of utilizing this information without the bias of outside websites is critically important as it becomes a tool used by millions of patients around the globe.

The primary objective of this study was to investigate whether ChatGPT-3.5 is a reliable source of peer-reviewed information for patients inquiring about total knee arthroplasty (TKA) and total hip arthroplasty (THA) procedures. Specifically, this is the first and only study to evaluate peer-reviewed sources used by ChatGPT including characteristics such as level of evidence and respective journal impact factor. Our hypothesis was that ChatGPT will provide reliable information and prioritize papers of higher levels of evidence, more recent dates of publication, and journals with higher impact factors. A secondary objective of this study was to investigate whether ChatGPT is more likely to utilize sources from journals based in the United States and/or OA journals to observe whether geographic- or industry-based biases exist, respectively. If found to be a reliable source of information for patients with the rigorous peer-reviewed literature informing its responses, ChatGPT may serve as an efficacious resource for prospective TKA and THA patients.

## 2. Methods

A retrospective analysis of ChatGPT's responses to preoperative patient questions, formulated based on common questions posed by patients in the clinic setting of various complexities and nuance in the literature, was conducted using the OA ChatGPT-3.5 most likely to be used by patients. Questions were formulated by the authors based on common questions asked in the clinic setting to challenge ChatGPT to critically assess the literature. These questions were posed to ChatGPT on November 2, 2023.

ChatGPT will not use the peer-reviewed literature to answer questions by default and must be directly asked by the user. However, once prompted to utilize the peer-reviewed literature, ChatGPT will adjust its methods to use the peer-reviewed literature for all subsequent questions. To address this phenomenon, the first question for TKA was phrased as “Utilizing only peer-reviewed literature, what options are available to treat knee arthritis besides surgery?” TKA and THA questions were asked in separate, otherwise blank conversations. ChatGPT used in-text citations in parentheses format and listed its corresponding references below each response, eliminating the need to ask ChatGPT which sources were used.

The first step was to determine whether the sources were accurate to existing publications, as recent sources warn that ChatGPT may provide false academic references [3]. To verify source accuracy, each was queried on PubMed, Scopus, Cochrane, and Google Scholar. If an article was present on any of these resources, its accuracy was confirmed by matching the authors, title, journal name, year of publication, and digital object identifier (DOI) numbers to what ChatGPT provided. Sources were only deemed accurate if all information provided by ChatGPT matched what was listed.

Each accurate source was categorized by the following factors: year of publication, journal name, journal impact factor, whether the journal is based in the United States or internationally, whether the journal is OA, the type of study in the source (randomized control trial, systematic review, and case series), and associated level of evidence. The clarivate analytics factor tool was used to identify the impact factors of each journal. The level of evidence for each source was appraised by the authors utilizing the Oxford Center for Evidence-Based Medicine 2011 Levels of Evidence guide.

Validity of responses was conducted via appraisal by the authors on a 6-level Likert scale for medical accuracy and a 3-level Likert scale for completeness. Discrepancies among evaluators were not addressed given our report of response averages rather than consensus for each response. Descriptive statistics were utilized to analyze the results of the accurate source characterization and validity. Our Institutional Review Board waived the need for approval for this study.

## 3. Results

Full transcripts of TKA and THA conversations are included in Supporting files 1 and 2, respectively, and an example response is included in Table 1. In response to 17 questions regarding TKA procedures, ChatGPT provided 56 total sources, 14 (25%) of which were deemed accurate. One of the accurate sources was utilized to answer multiple questions, yielding a total of 13 (23%) unique accurate sources. In response to the 16 questions regarding THA procedures, ChatGPT provided 53 total sources, 25 (47%) of which were deemed accurate. Three of these sources were utilized to answer multiple questions, yielding 22 (42%) unique sources. Overall, 39 sources (36%) were deemed accurate. Removing duplicate sources yielded 32 sources which are further characterized in their respective categories for TKA and THA questions in Tables 2 and 3, respectively.

**Figure 1:**
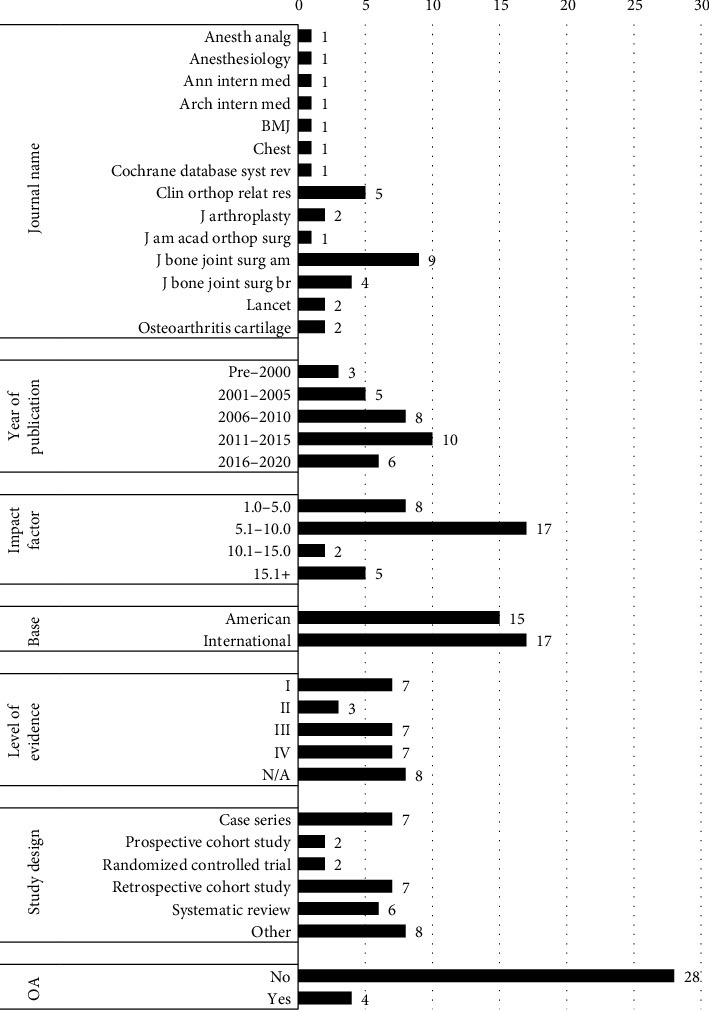
Characterization of accurate sources used by ChatGPT in response to TKA and THA questions.

Among accurate sources, the most cited journals were The Journal of Bone and Joint Surgery American Volume, Clinical Orthopedics and Related Research, and The Journal of Bone and Joint Surgery British Volume, with nine (28%), five (16%), and four (13%) sources, respectively (Figure 1). Seventeen sources (53%) came from journals based internationally, and 15 (47%) came from journals based in the United States. Eight (25%) sources came from journals with impact factors between 1.0 and 5.0, 17 (41%) sources came from journals with impact factors between 5.1 and 10.0, two (6%) sources came from journals with impact factors between 10.1 and 15.0, and five (17%) sources came from journals with impact factors above 15.0. Four (13%) sources were published in OA journals, while 28 (87%) were not.

The most common publication year bracket was 2011–2015 with 10 (31%) sources, followed by 2006–2010 with eight (25%) sources, 2016–2020 with six (19%) sources, 2001–2005 with five (16%) sources, and pre-2000 with three (8%) sources. Regarding levels of evidence, eight (25%) sources represented article types outside the traditional scale, including consensus statements, epidemiological studies, and projection studies. There were seven (22%) sources each in Levels I, III, and IV, and three (9%) sources in Level II. Regarding study designs, seven (22%) sources were retrospective cohort studies, seven (22%) sources were case series studies, six (19%) were systematic reviews, two (6%) were randomized controlled trials, and two (6%) were prospective cohort studies.

**Figure 2:**
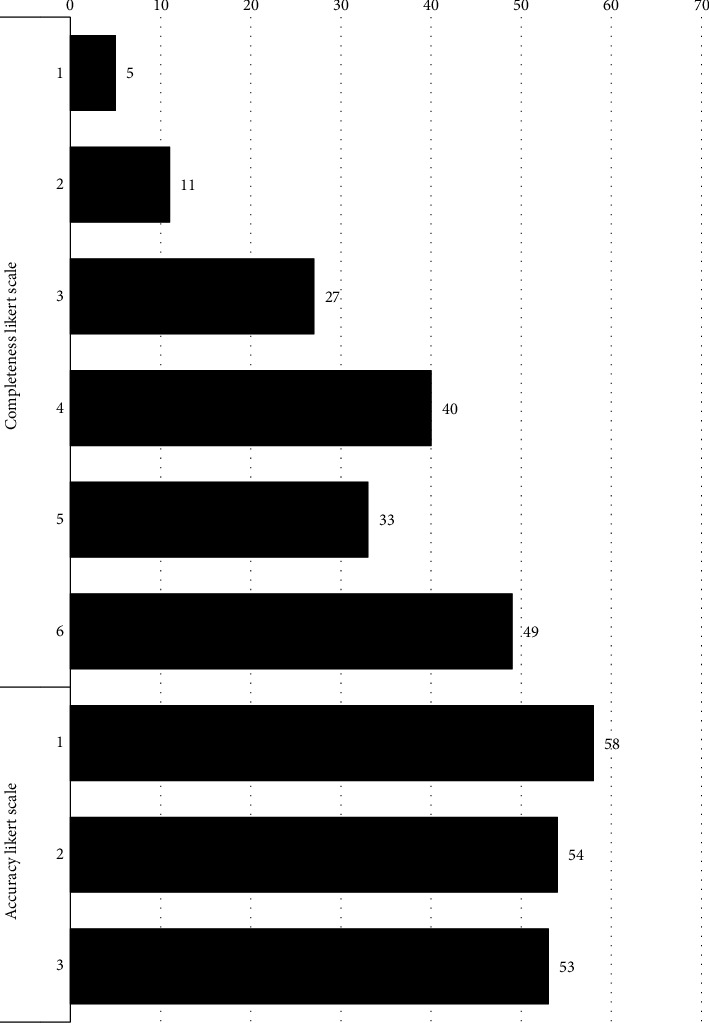
Surgeon appraisal of ChatGPT response to TKA and THA questions.

Evaluating ChatGPT responses found a combined average of 4.4 ± 1.4/6 on the Likert scale for accuracy which represents a value between “more correct than incorrect” and “nearly all correct” and a combined average of 1.92 ± 0.816/3 on the Likert scale for completeness which represents a value closest to “adequate, addresses all aspects of the question and provides the minimum amount of information required to be considered complete” (Figure 2).

## 4. Discussion

The present study sought to quantify and characterize peer-reviewed sources of evidence utilized by ChatGPT in response to common preoperative questions for TKA and THA. Reports have surfaced that ChatGPT is prone to utilizing unreliable sources, but this has not been characterized or quantified [10, 11]. The primary strength of this study is its novelty. To our knowledge, this is the first and only study in the literature to evaluate the peer-reviewed literature that ChatGPT uses to formulate its responses to common preoperative TKA and THA questions.

This study indicates that ChatGPT is an unreliable source of the peer-reviewed literature regarding questions related to TKA and THA as 64% of sources were deemed inaccurate, specifically referring to these sources as not able to be traced back to an existing publication. This finding is concerning as patients may be falsely informed ChatGPT. Medical researchers must be aware that there remains a need to externally validate all information and sources provided by ChatGPT [11–13].

These findings raise the question of how ChatGPT utilizes the source material to generate responses. If ChatGPT cites a piece of information with an accurate source, it stands to reason that it used that source's content to inform the response. In other words, ChatGPT was trained with the accurate source's contents and therefore provided reliable information to the user. However, when ChatGPT utilizes an inaccurate source to cite a piece of information, several processes may be responsible: (i) ChatGPT was trained with the correct information for its response outside of the peer-reviewed literature and created a fake source to adhere to the user input to cite peer-reviewed sources; (ii) ChatGPT is correctly utilizing the peer-reviewed literature but is unable to appropriately report all relevant information including authors, publication date, and DOI; or (iii) since ChatGPT is programed to use the information used to train it to create human-sounding responses, it may provide any combination of false information and false sources. These potential processes question the legitimacy of everything ChatGPT provides, even in cases where it utilizes accurate sources to provide valid responses.

ChatGPT is only capable of providing responses with the information it was trained with, and the current model is only trained with information through 2021 as of November, 2023. However, newer models, including ChatGPT-4.0, are able to access real-time Internet sources to formulate responses and may provide more reliable information. Nevertheless, there were fewer sources published between 2016 and 2020 compared with 2011 and 2015, and 2006 and 2010. These findings may indicate that rather than selectively weighing the qualities of potential sources to formulate a response, ChatGPT may be rapidly outputting the first relevant information being provided to it through an algorithm that does its best to meet the goals mentioned earlier.

The present findings indicate that ChatGPT does not necessarily prioritize the utilization of sources from higher impact factor journals, as 66% were found to come from journals with impact factors between 1.0 and 10, specifically with the highest quantity of accurate sources coming from journals with impact factors between 5.1 and 10, with 17, and the second highest quantity from journals with impact factors between 1.0 and 5.0, with eight. This finding is not unreasonable, as the focused subject material of respected orthopedic surgery journals including The Journal of Bone and Joint Surgery and Clinical Orthopedics and Related Research renders them to have lower impact factor ceilings when compared to larger journals such as The Lancet.

An ancillary objective of this study was to determine whether ChatGPT prioritizes sources from journals that are based in the United States or abroad and/or OA journals to investigate whether OpenAI's United States infrastructure might lead to information training with a geographic bias or, in the case of OA status, bias toward certain types of industry. This study found that with 17 sources coming from international journals and 15 coming from the United States journals, there is likely not a geographic bias. Four accurate sources came from OA journals, which carry several potential implications based on the nature of the two varieties. For one, patients utilizing ChatGPT with consideration of the peer-reviewed literature might predominately be offered references that are inaccessible to them. There also exist a range of potential biases and inclusion variations between what non-OA journals have a higher likelihood of publishing, potentially limiting the scope of ChatGPT's knowledge base. OA journals are also typically able to provide instant access to their published research in contrast to the often-longer publication protocols of non-OA journals. Future iterations of ChatGPT, such as the newer ChatGPT-4.0, that can survey the online landscape in real time may be impacted by this variation in recency.

Given the relatively recent advent, growth, and adoption of LLM technologies, clinicians are unlikely to have received education on how to practically approach this topic. Clinicians nevertheless must have an adequate understanding of the proper uses for AI technologies including ChatGPT to best inform patients. This study may guide clinicians in educating patients about the limitations of these technologies. Specifically, clinicians may utilize this study to communicate that ChatGPT's tendency to fabricate peer-reviewed evidence is salient and troublesome given that the adoption of medical knowledge from a clinician perspective is predicated in the literature published following rigorous peer review. Furthermore, even if ChatGPT is capable of providing generally accurate medical information, there currently appears to be no reliable method to delineate what is objectively true rather than fabricated for the purpose of providing a coherent humanoid response. Clinicians should thus advocate for patients to have extreme caution when utilizing these technologies and to verify ChatGPT's responses themselves with known reputable sources.

The generalizability of this study is notably limited by the specific request for ChatGPT to inform its responses with peer-reviewed sources while real-world prompts from patients are unlikely to include this stipulation. As ChatGPT may utilize any number of source types of varying academic integrity, ranging from scientific papers and reputable orthopedic organization websites to less reliable Wikipedia pages, news articles, and blogs, the responses evaluated in the present study may be dissimilar from what a patient may encounter. However, given the relatively reliable information found in this study's results despite the majority of inaccurate sources, it is unclear whether the information appraised here would be substantially different than real-world usage. Future studies should investigate this potential discrepancy between responses with or without instructions to utilize specific sources of information to more adequately understand what types of responses are possible with ChatGPT and how clinicians may more comprehensively advise patients on its usage.

This study is also limited in scope due to its limited sample size of 33 questions among TKA and THA procedures with 109 total sources provided. As ChatGPT is limited to the source material it was trained with, it is possible that other fields and domains may have more appropriately trained ChatGPT or vice versa. ChatGPT may provide unique responses to the same questions, especially if within the context of a discussion, and the responses are limited by the time of data collection. This study is inherently limited to the questions subjectively posited by the authors decreasing the generalizability of these results. Furthermore, using one AI tool among the various LLMs available and restricting prompts to peer-reviewed articles limits the generalizability of these responses to other uses of AI and the reliability of response validity given that patients will not typically ask specifically for peer-reviewed sources and thus receive different types of responses.

Regarding the validity component of this study, questions and responses were given to fellowship-trained orthopedic surgeons who were unblinded to the subject of the study and were not asked to compare responses to any specific reference materials, introducing potential subjectivity and bias. Future studies may have evaluations of ChatGPT responses conducted more objectively to obtain a better understanding of the medical accuracy and completeness of responses.

## 5. Conclusion

Recent advancements in AI and machine learning have prompted the development of language models such as ChatGPT. With its exponential rise in public attention, number of registered users, and a simple, human-sounding interface that can integrate various sources into one response, ChatGPT may be a source of information for patients seeking medical advice. The present study sought to investigate whether ChatGPT provides valid responses to common preoperative TKA and THA questions using peer-reviewed references and to characterize those sources. Despite its self-proclaimed attention to detail in appraising what sources to use in formulating its responses, only 35.78% of 109 total sources were deemed accurate. Further characterization of these sources revealed that most sources were published in 2011–2015 and from non-OA journals including The Journal of Bone and Joint Surgery. The most common study designs were retrospective cohort studies and case series studies with an equal distribution of sources with Levels of Evidence I, III, and IV. While ChatGPT may be a relatively reliable source of preoperative TKA and THA question responses, extreme caution must be taken to ensure whether that information came from accurate sources. Future iterations of ChatGPT may benefit from real-time Internet searchability and algorithms to ensure that only accurate sources may be referenced.

## Figures and Tables

**Table 1:** ChatGPT example response to a common THA question.

User	ChatGPT
Using only peer-reviewed literature, what options are available to treat hip arthritis besides surgery?	Hip arthritis is a degenerative joint disease that affects a significant proportion of the elderly population, causing pain and functional impairment. While hip replacement surgery is a common treatment for end-stage hip arthritis, there are several nonsurgical options available to manage the symptoms of hip arthritis. Here are some of the most commonly recommended nonsurgical treatments for hip arthritis based on the peer-reviewed literature: Physical therapy: A systematic review of randomized controlled trials (RCTs) found that physical therapy is effective in reducing pain and improving physical function in patients with hip osteoarthritis (OA) (1). Physical therapy interventions typically include exercise programs aimed at strengthening the hip and improving the range of motion. Non–steroidal anti-inflammatory drugs (NSAIDs): NSAIDs are commonly used to manage the pain and inflammation associated with hip arthritis. A systematic review of RCTs found that NSAIDs are effective in reducing pain and improving physical function in patients with hip OA (2). However, long-term use of NSAIDs can have adverse effects on gastrointestinal, cardiovascular, and renal function. Intra-articular corticosteroid injections: Intra-articular corticosteroid injections are a common treatment for hip arthritis. A systematic review of RCTs found that corticosteroid injections are effective in reducing pain and improving physical function in patients with hip OA (3). However, the effects are typically short-lived and repeat injections may be required. Glucosamine and chondroitin: Glucosamine and chondroitin are commonly used as dietary supplements for the treatment of hip arthritis. A systematic review of RCTs found that glucosamine and chondroitin are effective in reducing pain and improving physical function in patients with hip OA (4). However, the evidence is mixed, and the quality of the studies is variable. Weight loss: Obesity is a risk factor for the development of hip arthritis and can exacerbate the symptoms. A systematic review of RCTs found that weight loss is effective in reducing pain and improving physical function in patients with hip OA (5). Weight loss can be achieved through a combination of dietary modifications and exercise. References: Fransen M, McConnell S, Harmer AR, Van der Esch M, Simic M, Bennell KL. Exercise for osteoarthritis of the hip. Cochrane Database Syst Rev. 2014; 2014 (4):CD007912. Zhang W, Moskowitz RW, Nuki G, Abramson S, Altman RD, Arden N, Bierma-Zeinstra S, Brandt KD, Croft P, Doherty M, et al. OARSI recommendations for the management of hip and knee osteoarthritis, Part II: OARSI evidence-based, expert consensus guidelines. Osteoarthritis Cartilage. 2008; 16 (2):137–162. da Costa BR, Reichenbach S, Keller N, Nartey L, Wandel S, Juni P, Trelle S. Effectiveness of non-steroidal anti-inflammatory drugs for the treatment of pain in knee and hip osteoarthritis: a network meta-analysis.Lancet. 2017; 390 (10090):e21-e33. Towheed TE, Maxwell L, Anastassiades TP, et al. Glucosamine and chondroitin for the treatment of osteoarthritis: a systematic review. JAMA. 2000; 283 (11):1469–1475. Christensen R, Astrup A, Bliddal H. Weight loss: the treatment of choice for knee osteoarthritis? A randomized trial. Osteoarthritis Cartilage. 2005 Jan; 13 (1):20–7. doi: 10.1016/j.joca.2004.10.008.

**Table 2:** Unique accurate sources used by ChatGPT in response to total knee arthroplasty questions.

Source	Publication year	Journal	Journal location	Impact factor	Open access	Level of evidence	Study type
Uthman OA, et al.	2013	BMJ	International	96.216	No	I	Systematic review with meta-analysis
da Costa BR, et al.	2017	Lancet	International	202.731	Yes	I	Systematic review with meta-analysis
Bannuru RR, et al.	2015	Ann intern med	American	51.598	No	I	Systematic review with meta-analysis
Kurtz S, et al.	2007	J bone joint surg am	American	6.558	No	N/A	Projection
Fehring TK, et al.	2001	Clin orthop relat res	American	4.837	No	IV	Case series
Lonner JH, et al.	2000	Clin orthop relat res	American	4.837	No	IV	Case series
Meneghini RM, et al.	2008	J bone joint surg am	American	6.558	No	IV	Prospective case series
Parvizi J, Hanssen AD, et al.	2004	J bone joint surg am	American	6.558	No	IV	Case series
Choong PF, et al.	2009	J arthroplasty	International	4.435	No	I	Randomized controlled trial
Parvizi J, Stuart MJ, et al.	2001	Clin orthop relat res	American	4.837	No	IV	Case Series
Falck-Ytter Y, et al.	2012	Chest	American	11.393	No	N/A	Guidelines
Parvizi J, Huang R, et al.	2017	J bone joint surg am	American	6.558	No	II	Prospective cohort study
Warwick D, et al.	2007	J bone joint surg br	International	6.558	No	III	Retrospective cohort study

**Table 3:** Unique accurate sources used by ChatGPT in response to total hip arthroplasty questions.

Source	Publication year	Journal	Journal location	Impact factor	Open access?	Level of evidence	Study type
Fransen M, et al.	2014	Cochrane database syst rev	International	11.874	Yes	I	Systematic review
Zhang W, et al.	2008	Osteoarthritis cartilage	International	7.507	No	N/A	Guidelines
da Costa BR, et al.	2017	Lancet	International	202.731	Yes	I	Systematic review with meta-analysis
Christensen R, et al.	2005	Osteoarthritis cartilage	International	7.507	No	II	Randomized controlled trial
Zmistowski B, et al.	2013	J bone joint surg am	American	6.558	No	III	Retrospective cohort study
Warwick D, et al.	2007	J bone joint surg br	International	6.558	No	III	Retrospective cohort study
Bozic KJ, et al.	2009	J bone joint surg am	American	6.558	No	N/A	Epidemiological study
Barrack RL, et al.	1992	J bone joint surg br	International	6.558	No	IV	Case series
Engh CA Jr, et al.	1987	J bone joint surg br	International	6.558	No	III	Retrospective cohort study
Schmalzried TP, et al.	1992	J bone joint surg am	American	6.558	No	IV	Case series
Smith AJ, et al.	2012	Lancet	International	202.731	Yes	III	Retrospective cohort study
Bozic KJ, et al.	2009	J bone joint surg am	American	6.558	No	N/A	Epidemiological study
Kurtz SM, et al.	2011	Clin orthop relat res	American	4.837	No	I	Systematic review
Falck-Ytter, et al.	2012	Chest	American	11.393	No	N/A	Guidelines
Berger, R. A., et al.	2014	J bone joint surg am	American	6.558	No	II	Prospective cohort study
Langton, D. J., et al.	2009	J bone joint surg br	International	6.558	No	III	Retrospective cohort study
Kurtz, S. M., et al.	2019	Clin orthop relat res	American	4.837	No	N/A	Projection
Lalmohamed, A., et al.	2017	Arch intern med	International	51.598	Yes	III	Retrospective cohort study
Parvizi, J., Gehrke, T., Chen, A. F., et al.	2018	J arthroplasty	International	4.435	No	N/A	Consensus statement
Parvizi, J., & Gehrke, T.	2016	J am acad orthop surg	American	4	No	N/A	Guidelines
Memtsoudis, S. G., et al.	2013	Anesthesiology	International	9.198	No	III	Retrospective cohort
Guay, J., et al.	2014	Anesth analg	International	6.627	No	I	Systematic review

## Data Availability

The ChatGPT data used to support these findings can be found in the supporting files which include full conversation transcripts. No other data sources were utilized.

## Supporting Information

Full transcripts of TKA and THA conversations are included in supporting files 1 and 2, respectively.
