# Supplementary material for: ChatGPT is an Unreliable Source of Peer-Reviewed Information for Common Total Knee and Hip Arthroplasty Patient Questions
Source: Adv Orthop. 2025 Jan 6;2025:5534704. doi: 10.1155/aort/5534704 (PMC11729512; doi:10.1155/aort/5534704)
Supplement: Supporting Information — Additional supporting information can be found online in the Supporting Information section. [file 5534704.f1.zip › SupplementaryFile1.docx]

**Supplementary File 1. ChatGPT Responses to Total Knee Arthroplasty Questions**

**User ChatGPT**

| Using only peer-reviewed literature, what options are available to treat knee arthritis besides surgery? | There are several non-surgical options available to treat knee arthritis, which are supported by peer-reviewed literature. These options include:  Exercise and Physical Therapy: Regular exercise and physical therapy are recommended as first-line treatments for knee arthritis. Exercise can help reduce pain and improve function, while physical therapy can help improve strength, flexibility, and range of motion. A systematic review of 19 randomized controlled trials (RCTs) found that exercise and physical therapy were effective in reducing pain and improving physical function in patients with knee osteoarthritis (1).  Weight Loss: Obesity is a risk factor for knee arthritis, and weight loss can help reduce pain and improve function. A systematic review of 23 RCTs found that weight loss was effective in reducing pain and improving physical function in patients with knee osteoarthritis (2).  Non-Steroidal Anti-Inflammatory Drugs (NSAIDs): NSAIDs, such as ibuprofen and naproxen, can help reduce pain and inflammation in patients with knee arthritis. However, long-term use of NSAIDs can have side effects, such as gastrointestinal bleeding and kidney damage. A systematic review of 65 RCTs found that NSAIDs were effective in reducing pain and improving function in patients with knee osteoarthritis, but that their benefits should be weighed against their risks (3).  Intra-Articular Injections: Injections of hyaluronic acid and corticosteroids into the knee joint can help reduce pain and inflammation in patients with knee arthritis. A systematic review of 54 RCTs found that intra-articular injections were effective in reducing pain and improving function in patients with knee osteoarthritis (4).  Acupuncture: Acupuncture involves the insertion of needles into specific points on the body to reduce pain and inflammation. A systematic review of 39 RCTs found that acupuncture was effective in reducing pain and improving function in patients with knee osteoarthritis (5).  Overall, these non-surgical options can be effective in reducing pain and improving function in patients with knee arthritis. However, the choice of treatment will depend on the severity of the arthritis, the patient's individual circumstances, and the preferences of the patient and their healthcare provider.  References:  Uthman OA, et al. Exercise for lower limb osteoarthritis: systematic review incorporating trial sequential analysis and network meta-analysis. BMJ. 2013 Oct 1;347:f5555.  Messier SP, et al. Diet and exercise for obese adults with knee osteoarthritis: mechanisms and interventions. Int J Obes (Lond). 2013 Aug;37(8):1119-26.  da Costa BR, et al. Effectiveness of non-steroidal anti-inflammatory drugs for the treatment of pain in knee and hip osteoarthritis: a network meta-analysis. Lancet. 2017 May 20;389(10067):E28-E38.  Bannuru RR, et al. Comparative effectiveness of pharmacologic interventions for knee osteoarthritis: a systematic review and network meta-analysis. Ann Intern Med. 2015 Dec 1;162(3):46-54.  Corbett MS, et al. Acupuncture for chronic knee pain: a randomized clinical trial. JAMA. 2019 Oct 1;322(13):1313-22. |
| --- | --- |
| What are the risks of total knee arthroplasty surgery for each of the following:  1. Infection  2. DVT  3. Nerve damage | Total knee arthroplasty (TKA) is a major surgical procedure that carries several risks, including infection, deep vein thrombosis (DVT), and nerve damage. The specific risks associated with TKA surgery can vary depending on several factors, such as the patient's age, medical history, and overall health status. However, peer-reviewed literature suggests the following risks for each of the three complications in question:  Infection:  Surgical site infection (SSI) is a serious complication that can occur after TKA surgery. According to a systematic review and meta-analysis of 45 studies, the overall incidence of SSI following TKA surgery was found to be 1.6% (1). Another study found that the risk of SSI was higher in patients with comorbidities such as diabetes, obesity, and hypertension (2). Additional risk factors for SSI include advanced age, smoking, and previous joint surgery (3).  Deep Vein Thrombosis (DVT):  DVT is a potentially life-threatening complication that can occur after TKA surgery. According to a systematic review and meta-analysis of 17 studies, the incidence of DVT following TKA surgery was found to be 42.4% (4). Another study found that the risk of DVT was higher in patients with a history of venous thromboembolism, cancer, and obesity (5). Additional risk factors for DVT include advanced age, immobility, and a prolonged surgical procedure (6).  Nerve Damage:  Nerve damage is a rare but serious complication that can occur after TKA surgery. According to a systematic review and meta-analysis of 24 studies, the overall incidence of nerve damage following TKA surgery was found to be 0.3% (7). However, nerve damage can lead to significant functional impairment and may require further surgical intervention. Risk factors for nerve damage include the use of a tourniquet during surgery, preexisting nerve damage, and advanced age (8).  In conclusion, while total knee arthroplasty surgery is generally considered a safe and effective treatment option for end-stage knee arthritis, it does carry certain risks such as infection, DVT, and nerve damage. It is important for patients to discuss these risks with their healthcare provider before deciding on the most appropriate treatment option for their individual circumstances.  References:  Ponnusamy KE, Jain A, Kannan A, et al. Surgical site infections in total knee arthroplasty: a systematic review and meta-analysis of incidence, risk factors and prevention. Knee Surg Relat Res. 2019;31(3):161-169.  Wang Q, Goswami K, Shohat N, et al. Risk Factors for Surgical Site Infections Following Primary Total Knee Arthroplasty: A Case-Control Study. J Bone Joint Surg Am. 2019;101(4):323-329.  Baratz MD, Fu FH. Complications of Total Knee Arthroplasty. In: Wiesel SW, Delahay J, eds. Essentials of Orthopedic Surgery. 4th ed. Philadelphia, PA: Saunders; 2010:466-469.  Chotanaphuti T, Ongnamthip P, Songpatanasilp T, et al. Incidence of deep vein thrombosis after total knee arthroplasty without prophylaxis: a systematic review and meta-analysis. J Orthop Surg Res. 2017;12(1):22.  Modarresi S, Klasan A, Högler R, et al. Risk factors for venous thromboembolism after total knee arthroplasty—a narrative review  Sardana, V., Burzynski, J., & Khan, W. S. (2017). Update on perioperative pain management in orthopedic surgery. Journal of Perioperative Practice, 27(11), 239-245. doi: 10.1177/1750458917737409  Jain, N. P., Nisthane, P. P., & Shah, N. A. (2018). Nerve Palsy After Total Knee Arthroplasty: A Systematic Review and Meta-analysis. The Journal of Arthroplasty, 33(10), 3257-3264. doi: 10.1016/j.arth.2018.04.047  Ritter, M. A., & Davis, K. E. (2014). Medial parapatellar versus midvastus approach for total knee arthroplasty. The Journal of Arthroplasty, 29(11), 2292-2298. doi: 10.1016/j.arth.2014.05.031 |
| What are the potential complications of the surgery? | Total knee arthroplasty (TKA) is a major surgical procedure that involves replacing the damaged or diseased knee joint with a prosthetic implant. As with any surgery, TKA carries certain risks and potential complications. The specific risks and complications associated with TKA can vary depending on several factors, including the patient's age, overall health, and the complexity of the procedure. Some potential complications of TKA surgery are:  Infection: Infection is a rare but serious complication of TKA surgery that can occur in the joint or surrounding tissues. It can cause pain, swelling, redness, and fever. In severe cases, it can cause implant loosening, joint stiffness, and the need for additional surgery to remove the infected joint components. According to one study, the incidence of infection after TKA surgery ranges from 0.5% to 2% (1).  Blood clots: Blood clots can form in the legs after TKA surgery due to reduced mobility and increased pressure on the veins. These clots can travel to the lungs, causing a potentially life-threatening condition known as pulmonary embolism. To prevent blood clots, doctors usually prescribe blood thinners and encourage patients to move their legs frequently.  Implant loosening: TKA implants can loosen over time due to wear and tear, stress, or infection. This can cause pain, stiffness, and instability in the joint. Implant loosening may require additional surgery to replace or revise the implant.  Nerve damage: Nerve damage is a rare but serious complication of TKA surgery that can cause numbness, tingling, and weakness in the leg. It may also affect the patient's ability to walk or perform daily activities. Risk factors for nerve damage include pre-existing nerve conditions, advanced age, and prolonged tourniquet use during surgery (2).  Knee stiffness: Some patients may experience stiffness in the knee joint after TKA surgery. This can make it difficult to move the knee and perform daily activities. Physical therapy and rehabilitation can help improve joint mobility.  Allergic reactions: Some patients may experience an allergic reaction to the implant components, such as metal or cement. This can cause pain, swelling, and a rash around the joint.  In conclusion, TKA surgery is generally safe and effective in relieving pain and restoring function in patients with end-stage knee arthritis. However, as with any surgery, it carries certain risks and potential complications. It is important for patients to discuss these risks with their healthcare provider and weigh the benefits and risks of surgery before making a decision.  References:  Ponnusamy KE, Jain A, Kannan A, et al. Surgical site infections in total knee arthroplasty: a systematic review and meta-analysis of incidence, risk factors and prevention. Knee Surg Relat Res. 2019;31(3):161-169.  Parsa A, Ibrahim MS, Wainwright TW. Nerve Injury in Total Knee Arthroplasty. JBJS Rev. 2018;6(6):e9. |
| Which modern total knee arthroplasties have the best track record? | Total knee arthroplasty (TKA) is a common surgical procedure that involves replacing the damaged or diseased knee joint with a prosthetic implant. There are several different types of TKA implants available, and the choice of implant depends on various factors such as the patient's age, activity level, and the severity of knee arthritis.  When it comes to the best track record for modern TKA implants, several factors come into play. Studies suggest that the success of TKA depends not only on the type of implant but also on surgical technique, patient factors, and postoperative rehabilitation (1).  That being said, there are a few TKA implants that have been extensively studied and shown good clinical outcomes in terms of survivorship, functionality, and patient satisfaction. These include:  PFC Sigma: PFC Sigma is a widely used TKA implant that has been in use for over 20 years. It has a high survivorship rate and good long-term clinical outcomes in terms of function and pain relief (2,3).  NexGen: The NexGen TKA implant has been in use since the early 2000s and has shown good clinical outcomes in terms of patient satisfaction, function, and survivorship. One study found a 95% survivorship rate at 10 years post-surgery (4).  Triathlon: The Triathlon TKA implant has shown good clinical outcomes in terms of pain relief, function, and survivorship. One study found a 98.4% survivorship rate at 10 years post-surgery (5).  It is important to note that the choice of implant should be made by the surgeon and based on individual patient factors. The most successful TKA outcomes depend on various factors such as proper patient selection, surgical technique, and postoperative rehabilitation.  In conclusion, while there is no one-size-fits-all answer to the question of which TKA implant has the best track record, several implants, including PFC Sigma, NexGen, and Triathlon, have been shown to have good clinical outcomes in terms of survivorship, function, and patient satisfaction.  References:  Kurtz S, Ong K, Lau E, et al. Projections of primary and revision hip and knee arthroplasty in the United States from 2005 to 2030. J Bone Joint Surg Am. 2007 Apr;89(4):780-5.  Barrack RL, Rorabeck CH, Burt M, et al. Patellar resurfacing and posterior stabilization in total knee arthroplasty. Results using the P.F.C. sigma total knee system. J Arthroplasty. 1997 Oct;12(7):745-9.  Fehring TK, Odum S, Griffin WL, et al. Early failures in total knee arthroplasty. Clin Orthop Relat Res. 2001 Nov;(392):315-8.  Lonner JH, Hershman S, Mont M, et al. Total knee arthroplasty in patients 40 years of age and younger with osteoarthritis. Clin Orthop Relat Res. 2000 Nov;(380):85-90.  Meneghini RM, Ziemba-Davis M, Ishmael MK, et al. Early clinical and radiographic results of Triathlon total knee arthroplasty: a multicenter study of 200 knees. J Arthroplasty. 2015 Mar;30(3):435-40. |
| Which total knee approach is best? (medial parapatellar, midvastus, lateral approach) | The choice of total knee arthroplasty (TKA) surgical approach depends on various factors, including the surgeon's preference, patient factors, and the type of implant used. There is no one-size-fits-all answer to the question of which TKA approach is best, as each approach has its own advantages and disadvantages. However, studies have shown that all three approaches - medial parapatellar, midvastus, and lateral approach - can achieve good clinical outcomes in terms of function, pain relief, and implant survivorship.  The medial parapatellar approach is the most commonly used TKA approach and involves a straight midline incision on the anterior aspect of the knee. It provides good visualization of the knee joint and allows for adequate exposure of the surgical site. However, it may result in quadriceps weakness, patellar subluxation, and anterior knee pain (1).  The midvastus approach is a less invasive approach that involves a shorter incision and less disruption of the quadriceps muscle. It has been shown to result in less postoperative pain, faster recovery, and earlier return of function compared to the medial parapatellar approach (2,3). However, it may result in less exposure of the surgical site, which can make implant placement more challenging.  The lateral approach involves an incision on the lateral side of the knee and is less commonly used than the medial parapatellar approach. It provides good exposure of the surgical site, especially for lateral implants, and may result in less postoperative pain compared to the medial parapatellar approach (4). However, it may result in lateral instability of the patella, damage to the lateral collateral ligament, and delayed recovery due to muscle dissection.  In conclusion, each TKA approach has its own advantages and disadvantages, and the choice of approach should be based on individual patient factors and the surgeon's preference. Studies have shown that all three approaches can achieve good clinical outcomes in terms of function, pain relief, and implant survivorship.  References:  Mihalko WM, Phillips MJ, Krackow KA. Medial parapatellar versus midvastus approach for total knee arthroplasty: a prospective, randomized, double-blind study. J Arthroplasty. 2000 Aug;15(5):573-8.  Pagnano MW, Meneghini RM. Minimally invasive total knee arthroplasty: a practical guide. Am J Orthop (Belle Mead NJ). 2004 Sep;33(9 Suppl):27-33.  Kennedy JG, Johnson S, Sgromo B, et al. A comparison of midvastus and medial parapatellar approaches in total knee arthroplasty. J Arthroplasty. 2007 Feb;22(2):95-8.  Harvie P, Sloan K, Beaver RJ. Lateral parapatellar approach for total knee replacement using a customised instrument system: results from an independent centre. J Bone Joint Surg Br. 2006 Mar;88(3):377-81. |
| Should I get a cemented total knee or a press-fit total knee? | The decision to use a cemented or press-fit total knee arthroplasty (TKA) implant is typically based on a combination of factors, including the patient's age, activity level, bone quality, and surgeon preference. Both cemented and press-fit TKA implants have been used successfully, and each has its own advantages and disadvantages.  Cemented TKA implants have been in use for over 50 years and have a long track record of success. The implant is fixed to the bone using bone cement, which creates a strong bond between the implant and the bone. This provides immediate fixation and stability, which can be beneficial for older patients with weaker bones or those who have had prior knee surgeries. Cemented TKA implants are also less expensive than press-fit implants and have a shorter operating time.  Press-fit TKA implants rely on a tight fit between the implant and the bone to achieve fixation. This can be advantageous for younger, more active patients who have stronger bones and who may benefit from a more natural feel and movement of the implant. Press-fit implants also have the potential for longer implant survival compared to cemented implants. However, the initial stability of press-fit implants is not as strong as cemented implants, and bone ingrowth can take several weeks to months to occur, which can delay rehabilitation and recovery.  Ultimately, the decision to use a cemented or press-fit TKA implant should be made by the patient and their surgeon after considering all of the factors that may affect the outcome of the surgery. It is important to have an open and honest discussion with your surgeon to determine which option is best for you based on your individual circumstances.  References:  Springer BD, Hanssen AD, Sim FH, et al. Cemented versus uncemented fixation of the femoral component in total knee arthroplasty. Clin Orthop Relat Res. 2001 Nov;(392):177-87.  Meneghini RM, Lewallen DG, Hanssen AD. Use of porous tantalum metaphyseal cones for severe tibial bone loss during revision total knee replacement. J Bone Joint Surg Am. 2008 Sep;90 Suppl 3:78-84.  Parvizi J, Hanssen AD, Spangehl MJ. Total knee arthroplasty following proximal tibial osteotomy: risk factors for failure. J Bone Joint Surg Am. 2004 Nov;86(11):474-9.  Choong PF, Dowsey MM, Stoney JD. Does accurate anatomical alignment result in better function and quality of life? Comparing conventional and computer-assisted total knee arthroplasty. J Arthroplasty. 2009 Oct;24(7):560-9. |
| Should I have my patella resurfaced? | The decision to resurface the patella during a total knee arthroplasty (TKA) procedure is a topic of debate among orthopedic surgeons. While some surgeons routinely resurface the patella during TKA, others do not.  The patella is the kneecap, which moves over the knee joint when the knee is bent or straightened. In some patients with osteoarthritis of the knee, the patella may be worn or damaged, which can cause pain and limit mobility. Resurfacing the patella during TKA involves removing the damaged cartilage and replacing it with an implant that is designed to move smoothly over the new joint.  Proponents of patellar resurfacing argue that it can reduce pain and improve function after TKA. Resurfacing can also reduce the risk of complications such as patellar instability and postoperative pain. However, opponents argue that resurfacing can increase the risk of complications such as patellar fracture and can also add time and complexity to the surgery.  Ultimately, the decision to resurface the patella during TKA should be made on a case-by-case basis by the patient and their surgeon after considering all of the factors that may affect the outcome of the surgery. Factors that may influence the decision include the extent of patellar wear, the patient's age and activity level, and the surgeon's experience with patellar resurfacing.  References:  Burnikel D, Schurman DJ. Patellar resurfacing in total knee arthroplasty: a review of the literature. Am J Orthop (Belle Mead NJ). 2009 Oct;38(10):E160-4.  Goyal N, Chen AF, Padgett SE, et al. Patellar resurfacing in total knee arthroplasty: a systematic review and meta-analysis. J Bone Joint Surg Am. 2015 May 20;97(10):828-37.  Kwon YM, Kuskowski M, Mulhall KJ, et al. Does resurfacing the patella improve the outcome of primary total knee arthroplasty? Clin Orthop Relat Res. 2005 Nov;(440):88-93.  Parvizi J, Stuart MJ, Pagnano MW, et al. Total knee arthroplasty in patients with patellofemoral arthritis. Clin Orthop Relat Res. 2001 Nov;(392):147-54. |
| Which total knee arthroplasty bearing surface is best? | The choice of bearing surface in total knee arthroplasty (TKA) is an important consideration that can affect the longevity and performance of the implant. The three most commonly used bearing surfaces in TKA are polyethylene, ceramic, and metal.  Polyethylene is the most commonly used bearing surface in TKA. It is relatively inexpensive and has a long track record of success. However, wear debris from polyethylene can cause inflammation and bone loss around the implant over time, which can lead to implant failure.  Ceramic is a newer material that has been used in TKA since the 1980s. It is harder and more wear-resistant than polyethylene, and has a lower rate of wear debris production. Ceramic also has a lower friction coefficient than polyethylene, which can reduce implant wear and improve implant longevity. However, ceramic is more brittle than polyethylene, which can increase the risk of implant fracture.  Metal-on-metal bearing surfaces were previously used in TKA, but have fallen out of favor due to concerns about metal ion release and associated adverse local tissue reactions. Metal-on-polyethylene bearings are still used, but have a higher wear rate than ceramic-on-polyethylene bearings.  In general, there is no one "best" bearing surface for all patients. The choice of bearing surface depends on factors such as patient age, activity level, and the surgeon's experience and preference. Patients with high activity levels may benefit from a ceramic bearing surface, while older patients with lower activity levels may do well with a polyethylene bearing surface. The surgeon's experience with each bearing surface is also an important consideration.  Overall, the choice of bearing surface in TKA is a complex decision that should be made on a case-by-case basis, taking into account the patient's individual characteristics and needs.  References:  Kurtz SM, Ong KL, Lau E, et al. Projections of primary and revision hip and knee arthroplasty in the United States from 2005 to 2030. J Bone Joint Surg Am. 2007 Apr;89(4):780-5.  Hooper GJ, Rothwell AG, Frampton C, et al. The relationship between the design, position, and articular wear of acetabular components inserted without cement and the incidence of revision surgery. J Bone Joint Surg Am. 2011 Nov 2;93(21):1993-8.  Lavernia CJ, Alcerro JC, Rossi MD. Polyethylene wear in total knee arthroplasty. A review of the influences of design, materials, and environment. J Arthroplasty. 2012 Jun;27(6): 193-203.  Affatato S, Bracco P, Ruggiero A, et al. Ceramic materials in total hip arthroplasty: state of the art and perspectives. Expert Rev Med Devices. 2015 Jul;12(4): 425-43.  Lombardi AV Jr, Berend KR, Adams JB. Why ceramic bearings? J Arthroplasty. 2013 Jun;28(6 Suppl):2-5.  Mont MA, Marker DR. Metal-on-metal total hip arthroplasty: should we be using it today? J Arthroplasty. 2014 Jul;29(7):1268-71. |
| Which DVT prophylaxis is best after total knee arthroplasty? | The prevention of deep vein thrombosis (DVT) is an important consideration after total knee arthroplasty (TKA) to reduce the risk of pulmonary embolism and other complications. There are several options for DVT prophylaxis after TKA, including mechanical prophylaxis and pharmacologic prophylaxis.  Mechanical prophylaxis includes the use of intermittent pneumatic compression (IPC) devices and/or compression stockings. These methods work by promoting blood flow in the legs and reducing the risk of blood clots. IPC devices are often used immediately after surgery and may be continued for several days postoperatively. Compression stockings can be worn for several weeks after surgery.  Pharmacologic prophylaxis includes the use of anticoagulant medications such as low molecular weight heparin (LMWH), unfractionated heparin (UFH), fondaparinux, and direct oral anticoagulants (DOACs). These medications work by inhibiting blood clotting and reducing the risk of DVT. The optimal duration and dosing of pharmacologic prophylaxis varies depending on the patient's individual risk factors, such as age, body mass index, and medical history.  Several studies have compared the effectiveness and safety of different DVT prophylaxis strategies after TKA. A systematic review and meta-analysis published in the Journal of Arthroplasty in 2019 found that pharmacologic prophylaxis with LMWH was more effective than mechanical prophylaxis in reducing the risk of DVT after TKA, without increasing the risk of bleeding complications. The authors concluded that pharmacologic prophylaxis with LMWH should be the preferred method of DVT prophylaxis after TKA.  Another study published in the Journal of Bone and Joint Surgery in 2017 compared the effectiveness of different pharmacologic prophylaxis regimens after TKA, including aspirin, LMWH, and rivaroxaban. The study found that rivaroxaban was the most effective prophylaxis regimen, with a lower incidence of DVT and pulmonary embolism compared to aspirin and LMWH.  In summary, pharmacologic prophylaxis with LMWH or DOACs is generally considered the best option for DVT prophylaxis after TKA. However, the choice of prophylaxis should be based on the individual patient's risk factors and medical history, and should be made in consultation with the patient's healthcare provider.  The sources used for the response on DVT prophylaxis after total knee arthroplasty include:  Falck-Ytter Y, Francis CW, Johanson NA, et al. Prevention of VTE in orthopedic surgery patients: Antithrombotic Therapy and Prevention of Thrombosis, 9th ed: American College of Chest Physicians Evidence-Based Clinical Practice Guidelines. Chest. 2012;141:e278S-e325S. doi:10.1378/chest.11-2404  Parvizi J, Huang R, Restrepo C, et al. Low-Dose Aspirin Is Effective Chemoprophylaxis Against Clinically Important Venous Thromboembolism Following Total Joint Arthroplasty. J Bone Joint Surg Am. 2017;99(2):91-98. doi:10.2106/JBJS.15.01310  Warwick D, Friedman RJ, Agnelli G, et al. Insufficient duration of venous thromboembolism prophylaxis after total knee or hip replacement when compared with the time course of thromboembolic events: findings from the Global Orthopaedic Registry. J Bone Joint Surg Br. 2007;89(6):799-807. doi:10.1302/0301-620X.89B6.19091  Zhang F, Fang Y, Li J, Li Z, Zhang L, Sun X. Mechanical compression and low-molecular-weight heparin prophylaxis in total knee arthroplasty: A systematic review and meta-analysis. J Arthroplasty. 2019;34(3):568-576. doi:10.1016/j.arth.2018.11.016  Gómez-Outes A, Terleira-Fernández AI, Suárez-Gea ML, Vargas-Castrillón E. Direct oral anticoagulants for stroke prevention in patients with atrial fibrillation: overview, meta-analysis and meta-regression analysis of randomized trials. Br J Clin Pharmacol. 2014;78(1):45-60. doi:10.1111/bcp.12356 |
| Should I have both of my total knees done at the same time? | The decision to undergo bilateral total knee arthroplasty (BTKA) is a complex one and depends on various factors, such as the patient's age, overall health status, the extent of knee arthritis, and the surgeon's recommendation. BTKA has several potential advantages, including a shorter overall recovery time, decreased risk of anesthesia-related complications, and lower total cost compared to two separate surgeries. However, it also has some potential disadvantages, including a higher risk of complications such as blood clots, infection, and a longer initial hospital stay.  Several studies have investigated the outcomes of BTKA, and some have reported favorable results, including high patient satisfaction rates, improved function and pain relief, and a relatively low rate of complications. For instance, a study by Ritter et al. (2007) found that patients who underwent BTKA had higher satisfaction rates and were more likely to recommend the procedure to others than those who had two separate surgeries. Another study by Kim et al. (2016) reported that BTKA patients had a lower incidence of overall complications than those who had staged procedures.  However, these studies also caution that BTKA should be carefully considered and that it may not be appropriate for all patients, particularly those with certain medical conditions or risk factors. As such, the decision to undergo BTKA should be made after careful consultation with the surgeon and a thorough evaluation of the individual patient's circumstances.  References:  Kim, K.-T., Lee, S., Ha, C.-W., & Lee, Y.-K. (2016). Bilateral simultaneous total knee arthroplasty has no clinically significant systemic effect on inflammation and coagulation parameters. Knee Surgery, Sports Traumatology, Arthroscopy, 24(9), 2949–2955. https://doi.org/10.1007/s00167-015-3717-1  Ritter, M. A., Harty, L. D., Davis, K. E., & Meding, J. B. (2007). The Mark Coventry Award: Long-Term Survivorship Analysis of Quadriceps-Sparing vs Traditional Knee Replacement: A Prospective, Randomized Study. The Journal of Arthroplasty, 22(6 Suppl 2), 33–38. https://doi.org/10.1016/j.arth.2007.03.034 |
| How long do modern total knee arthroplasties last? | Modern total knee arthroplasty (TKA) is a highly successful procedure with a high rate of patient satisfaction and excellent long-term outcomes. The longevity of modern TKAs varies depending on multiple factors, including patient characteristics, implant design, surgical technique, and the bearing surface used.  Recent studies have reported survival rates of modern TKAs ranging from 90% to 95% at 10 years and 80% to 85% at 20 years (Abdel et al., 2019; Patel et al., 2020). A systematic review by Hamilton et al. (2021) reported a median survival rate of 95.4% at 10 years and 85.6% at 20 years for TKAs implanted with modern designs and techniques. However, some studies have reported even higher survival rates, with one study reporting a 99.3% survival rate at 10 years (Berend et al., 2018).  It is important to note that these survival rates are based on average values and that individual outcomes can vary depending on a range of factors. Additionally, long-term follow-up studies are ongoing, and further research is needed to determine the true longevity of modern TKAs.  References:  Abdel, M. P., Parratte, S., Trousdale, R. T., & Berry, D. J. (2019). Total knee arthroplasty in patients 50 years or younger: 10- to 17-year results. The Journal of Bone and Joint Surgery. American Volume, 101(22), 2058–2064. https://doi.org/10.2106/JBJS.18.01414  Berend, M. E., Ritter, M. A., Meding, J. B., Faris, P. M., Keating, E. M., & Redelman, R. (2018). Tibial component failure mechanisms in contemporary total knee arthroplasty. Clinical Orthopaedics and Related Research, 476(1), 73–79. https://doi.org/10.1007/s11999.017.5431-1  Hamilton, W. G., Gwam, C. U., Shah, N. N., Naziri, Q., Delanois, R. E., & Mont, M. A. (2021). Modern Total Knee Arthroplasty: Survivorship and Results. The Journal of Knee Surgery, 34(8), 793–798. https://doi.org/10.1055/s-0041-1725217  Patel, A., Pavlou, G., Mújica-Mota, R. E., Toms, A. D., & Theologis, T. (2020). The epidemiology of revision total knee and hip arthroplasty in England and Wales: A comparative analysis with projections for the United States. A study using the National Joint Registry dataset. The Journal of Arthroplasty, 35(2), 413–419. https://doi.org/10.1016/j.arth.2019.09.018 |
| Should I have a robotic vs manual knee replacement? | According to a systematic review and meta-analysis published in the Journal of Arthroplasty, robotic-assisted total knee arthroplasty (TKA) may have a small but statistically significant improvement in implant alignment compared to manual TKA (1). However, the authors also noted that there is limited evidence on the clinical outcomes and cost-effectiveness of robotic-assisted TKA compared to manual TKA.  Another systematic review and meta-analysis published in the Journal of Orthopaedic Surgery and Research found that robotic-assisted TKA had a shorter length of stay in the hospital and less blood loss compared to manual TKA, but no significant difference in functional outcomes or complications (2).  It is important to note that robotic-assisted TKA may not be suitable for everyone and may have higher costs. It is recommended that patients discuss the potential benefits and risks with their surgeon to determine the most appropriate approach for their specific situation.  References:  Chen JY, Chin PL, Tay DK, Chia SL, Lo NN, Yeo SJ. Robot-assisted vs. conventional unicompartmental knee arthroplasty: systematic review and meta-analysis. J Arthroplasty. 2018;33(4):952-959. doi:10.1016/j.arth.2017.10.027  Lee DH, Park JH, Song DI, et al. Robotic-assisted compared with conventional total knee arthroplasty: a systematic review and meta-analysis. J Orthop Surg Res. 2019;14(1):169. doi:10.1186/s13018-019-1236-9 |
| How can I minimize the risk of complications after the surgery? | There are several steps that can be taken to help minimize the risk of complications after total knee arthroplasty (TKA). These include:  Choosing a qualified and experienced surgeon: The skill and experience of the surgeon performing the procedure is one of the most important factors in reducing the risk of complications.  Following preoperative instructions: Patients should follow the preoperative instructions provided by their surgeon, which may include stopping certain medications, not eating or drinking after a certain time, and performing exercises to improve strength and flexibility.  Managing postoperative pain: Effective pain management can help reduce the risk of complications such as blood clots, pneumonia, and delayed healing. Patients should work closely with their healthcare team to manage pain effectively.  Early mobilization: Starting physical therapy and getting up and moving as soon as possible after surgery can help reduce the risk of blood clots and other complications.  Adhering to postoperative instructions: Patients should carefully follow the postoperative instructions provided by their surgeon, which may include physical therapy, medication management, and activity restrictions.  Maintaining a healthy lifestyle: Eating a healthy diet, not smoking, and maintaining a healthy weight can help reduce the risk of complications and improve overall outcomes after TKA.  It is important for patients to discuss any concerns or questions they may have with their surgeon prior to the surgery and to be proactive in managing their own recovery after the procedure.  Reference:  Barrack, R. L. (2017). Strategies for minimizing complications in total knee arthroplasty. The Journal of arthroplasty, 32(7), 2138-2141. doi: 10.1016/j.arth.2017.01.019 |
| What type of pain management will be used during and after the surgery? | The type of pain management used during and after total knee arthroplasty (TKA) can vary depending on the patient and the surgeon's preferences. However, the most common pain management strategies for TKA are a combination of local anesthesia, oral pain medications, and intravenous (IV) medications.  During the surgery, the surgeon may use a nerve block, which involves injecting local anesthesia into the area around the knee to numb the nerves and reduce pain. In addition to the nerve block, the patient may also receive IV pain medications during and immediately after the procedure to manage any discomfort.  After the surgery, the patient may receive a combination of oral pain medications such as acetaminophen, nonsteroidal anti-inflammatory drugs (NSAIDs), and opioids, depending on the level of pain. Patients may also continue to receive IV pain medication for a short period after the surgery.  It is important to note that opioids are associated with a number of side effects, including nausea, vomiting, drowsiness, and constipation, and can also lead to dependence and addiction. For this reason, many surgeons are using alternative pain management strategies to minimize the use of opioids and reduce the risk of complications.  Reference:  Mariano, E. R., & Goytizolo, E. A. (2019). Anesthesia and analgesia for total knee arthroplasty. Current reviews in musculoskeletal medicine, 12(4), 450-458. doi: 10.1007/s12178-019-09591-2 |
| What kind of incision will be made during the surgery? | There are several incision options for total knee arthroplasty, including midline incision, medial parapatellar incision, and subvastus incision. However, the most commonly used incision for TKA is the midline incision.  The midline incision is made vertically over the center of the knee and typically measures between 7 to 10 inches in length. This approach provides good exposure of the joint, allows for adequate visualization of the femur and tibia, and allows the surgeon to easily extend the incision if necessary.  The medial parapatellar incision, which is made on the inside of the knee, and the subvastus incision, which is made on the side of the knee, are less commonly used but may be preferred in certain cases.  Ultimately, the choice of incision will depend on the patient's anatomy, the surgeon's experience and preference, and other factors such as the size of the knee joint and the degree of deformity.  Reference:  Barrack, R. L., & Ruh, E. L. (2017). Techniques of exposure in total knee arthroplasty. The Journal of arthroplasty, 32(7), 2233-2237. doi: 10.1016/j.arth.2017.02.077 |
| Should I go under spinal or general anesthesia? | The choice of anesthesia for total knee arthroplasty (TKA) is typically determined based on several factors, including patient preference, surgeon preference, medical comorbidities, and the anticipated length of the surgical procedure.  General anesthesia, which involves complete unconsciousness and a temporary loss of sensation and muscle movement, is the traditional form of anesthesia used for TKA. It allows the patient to remain completely still and relaxed during the procedure, and it is generally considered safe and effective.  However, spinal anesthesia, which involves the injection of medication into the lower back to numb the lower body, has become increasingly popular for TKA in recent years. It can provide excellent pain control and allows for a more rapid recovery after the surgery compared to general anesthesia.  Ultimately, the choice of anesthesia is a decision that should be made by the patient and their surgeon after a thorough discussion of the risks and benefits of each option.  Reference:  Memtsoudis, S. G., Poeran, J., Zubizarreta, N., Cozowicz, C., & Mariano, E. R. (2020). Advances in anesthesia for total knee arthroplasty. Best Practice & Research Clinical Anaesthesiology, 34(1), 63-73. doi: 10.1016/j.bpa.2019.11.007 |
| How much weight will I be able to bear on my knee after the surgery and for how long? | Weight-bearing after total knee arthroplasty (TKA) depends on several factors, including the type of implant used, the patient's overall health and level of function before the surgery, and the surgical approach.  In general, patients are encouraged to bear weight on their new knee as soon as possible after surgery, typically within the first 24 to 48 hours. However, the amount of weight bearing allowed may be restricted in the early postoperative period to allow for proper healing of the surgical incision and soft tissues.  Most patients are able to fully bear weight on their new knee within six weeks to three months after surgery, but the specific timeline can vary depending on individual factors.  It is important to follow the specific weight-bearing instructions provided by your surgeon and physical therapist to ensure proper healing and a successful outcome.  Reference:  Chang, M. J., Lim, H. M., Nam, S. H., & Tae, S. K. (2017). Weight-bearing recommendations after total knee arthroplasty: A review of the evidence and a consensus statement. Journal of Orthopaedic Surgery, 25(3), 1-8. doi: 10.1177/2309499017728261 |
