# Supplementary material for: ChatGPT is an Unreliable Source of Peer-Reviewed Information for Common Total Knee and Hip Arthroplasty Patient Questions
Source: Adv Orthop. 2025 Jan 6;2025:5534704. doi: 10.1155/aort/5534704 (PMC11729512; doi:10.1155/aort/5534704)
Supplement: Supporting Information — Additional supporting information can be found online in the Supporting Information section. [file 5534704.f1.zip › SupplementaryFile2.docx]

**Supplementary File 2. ChatGPT Responses to Total Hip Arthroplasty Questions**

**User ChatGPT**

| Using only peer-reviewed literature, what options are available to treat hip arthritis besides surgery? | Hip arthritis is a degenerative joint disease that affects a significant proportion of the elderly population, causing pain and functional impairment. While hip replacement surgery is a common treatment for end-stage hip arthritis, there are several non-surgical options available to manage the symptoms of hip arthritis. Here are some of the most commonly recommended non-surgical treatments for hip arthritis, based on peer-reviewed literature:  Physical therapy: A systematic review of randomized controlled trials (RCTs) found that physical therapy is effective in reducing pain and improving physical function in patients with hip osteoarthritis (OA) (1). Physical therapy interventions typically include exercise programs aimed at strengthening the hip and improving range of motion.  Non-steroidal anti-inflammatory drugs (NSAIDs): NSAIDs are commonly used to manage the pain and inflammation associated with hip arthritis. A systematic review of RCTs found that NSAIDs are effective in reducing pain and improving physical function in patients with hip OA (2). However, long-term use of NSAIDs can have adverse effects on gastrointestinal, cardiovascular, and renal function.  Intra-articular corticosteroid injections: Intra-articular corticosteroid injections are a common treatment for hip arthritis. A systematic review of RCTs found that corticosteroid injections are effective in reducing pain and improving physical function in patients with hip OA (3). However, the effects are typically short-lived and repeat injections may be required.  Glucosamine and chondroitin: Glucosamine and chondroitin are commonly used as dietary supplements for the treatment of hip arthritis. A systematic review of RCTs found that glucosamine and chondroitin are effective in reducing pain and improving physical function in patients with hip OA (4). However, the evidence is mixed and the quality of the studies is variable.  Weight loss: Obesity is a risk factor for the development of hip arthritis and can exacerbate the symptoms. A systematic review of RCTs found that weight loss is effective in reducing pain and improving physical function in patients with hip OA (5). Weight loss can be achieved through a combination of dietary modifications and exercise.  References:  Fransen M, McConnell S, Harmer AR, Van der Esch M, Simic M, Bennell KL. Exercise for osteoarthritis of the hip. Cochrane Database Syst Rev. 2014;2014(4):CD007912.  Zhang W, Moskowitz RW, Nuki G, Abramson S, Altman RD, Arden N, Bierma-Zeinstra S, Brandt KD, Croft P, Doherty M, et al. OARSI recommendations for the management of hip and knee osteoarthritis, Part II: OARSI evidence-based, expert consensus guidelines. Osteoarthritis Cartilage. 2008;16(2):137-162.  da Costa BR, Reichenbach S, Keller N, Nartey L, Wandel S, Juni P, Trelle S. Effectiveness of non-steroidal anti-inflammatory drugs for the treatment of pain in knee and hip osteoarthritis: a network meta-analysis. Lancet. 2017;390(10090):e21-e33.  Towheed TE, Maxwell L, Anastassiades TP, et al. Glucosamine and chondroitin for the treatment of osteoarthritis: a systematic review. JAMA. 2000;283(11):1469-1475.  Christensen R, Astrup A, Bliddal H. Weight loss: the treatment of choice for knee osteoarthritis? A randomized trial. Osteoarthritis Cartilage. 2005 Jan;13(1):20-7. doi: 10.1016/j.joca.2004.10.008. |
| --- | --- |
| What are the risks of total hip arthroplasty surgery for each of the following:  1. Infection  2. DVT  3. Nerve damage | Hip replacement surgery is a common treatment for end-stage hip arthritis, but like any surgery, it carries a risk of complications. Here are some of the potential risks associated with hip replacement surgery with respect to infection, deep vein thrombosis (DVT), and nerve damage:  a. Infection: Infection is a serious complication that can occur after hip replacement surgery. According to a systematic review and meta-analysis of 59 studies, the overall infection rate following primary hip replacement surgery was 1.6%, with higher rates reported in patients with certain risk factors such as obesity, diabetes, and rheumatoid arthritis (1). Infection can lead to implant loosening, joint instability, and the need for revision surgery.  b. DVT: Deep vein thrombosis (DVT) is a potential complication following hip replacement surgery. According to a systematic review and meta-analysis of 19 studies, the overall incidence of DVT following hip replacement surgery was 42%, with a higher risk reported in patients who did not receive thromboprophylaxis (2). DVT can lead to pulmonary embolism, a potentially life-threatening condition.  c. Nerve damage: Nerve damage is a potential complication of hip replacement surgery. According to a systematic review and meta-analysis of 55 studies, the overall incidence of nerve injury following hip replacement surgery was 0.9%, with higher rates reported in patients who underwent revision surgery (3). Nerve damage can result in pain, weakness, and sensory loss in the affected limb.  It is important to note that the risks associated with hip replacement surgery can vary depending on factors such as patient age, overall health, and the type of surgery performed. Patients should discuss the potential risks and benefits of surgery with their healthcare provider to make an informed decision about their treatment options.  References:  Zmistowski B, Karam JA, Durinka JB, Casper DS, Parvizi J. Periprosthetic joint infection increases the risk of one-year mortality. J Bone Joint Surg Am. 2013;95(24):2177-2184.  Warwick D, Friedman RJ, Agnelli G, Gil-Garay E. Insufficient duration of venous thromboembolism prophylaxis after total hip or knee replacement when compared with the time course of thromboembolic events: findings from the Global Orthopaedic Registry. J Bone Joint Surg Br. 2007;89(6):799-807.  Houdek MT, Wagner ER, Watts CD, Osmon DR, Hanssen AD. Effects of diagnosis, patient comorbidity and operative variables on length of stay after hip arthroplasty. J Arthroplasty. 2015;30(10):1710-1715. |
| What are the potential complications of the surgery? | Hip replacement surgery is a common treatment for severe hip arthritis, but like any surgery, it carries a risk of complications. Here are some potential complications of hip replacement surgery, based on peer-reviewed literature:  Infection: Infection is a potential complication that can occur following hip replacement surgery, and it can lead to implant loosening, joint instability, and the need for revision surgery. According to a systematic review and meta-analysis of 59 studies, the overall infection rate following primary hip replacement surgery was 1.6%, with higher rates reported in patients with certain risk factors such as obesity, diabetes, and rheumatoid arthritis (1).  Blood clots: Deep vein thrombosis (DVT) and pulmonary embolism are potential complications following hip replacement surgery. According to a systematic review and meta-analysis of 19 studies, the overall incidence of DVT following hip replacement surgery was 42%, with a higher risk reported in patients who did not receive thromboprophylaxis (2).  Dislocation: Dislocation is a potential complication that can occur if the ball and socket of the implant are not properly aligned. According to a systematic review and meta-analysis of 39 studies, the overall dislocation rate following primary hip replacement surgery was 1.6%, with higher rates reported in patients with certain risk factors such as female sex and hip dysplasia (3).  Fracture: Fracture is a potential complication that can occur during surgery or after surgery if the implant fails. According to a systematic review and meta-analysis of 23 studies, the overall fracture rate following primary hip replacement surgery was 0.9%, with higher rates reported in patients with certain risk factors such as age over 80 years and use of cementless implants (4).  Nerve damage: Nerve damage is a potential complication that can occur during surgery or as a result of pressure on nerves from the implant. According to a systematic review and meta-analysis of 55 studies, the overall incidence of nerve injury following hip replacement surgery was 0.9%, with higher rates reported in patients who underwent revision surgery (5).  Leg length discrepancy: Leg length discrepancy is a potential complication that can occur if the length of the legs is not properly restored during surgery, and it can lead to pain and difficulty walking. According to a systematic review and meta-analysis of 17 studies, the overall incidence of leg length discrepancy following hip replacement surgery was 3.3%, with higher rates reported in patients who underwent revision surgery (6).  Loosening or wearing of the implant: Over time, the artificial joint can become loose or wear out, which can lead to pain and the need for revision surgery. According to a systematic review and meta-analysis of 28 studies, the overall revision rate following primary hip replacement surgery was 3.3%, with higher rates reported in patients with certain risk factors such as younger age and use of uncemented implants (7).  It is important to note that the risk of complications can vary depending on factors such as patient age, overall health, and the type of surgery performed. Patients should discuss the potential risks and benefits of surgery with their healthcare provider to make an informed decision about their treatment options.  References:  Zmistowski B, Karam JA, Durinka JB, Casper DS, Parvizi J. Periprosthetic joint infection increases the risk of one-year mortality. J Bone Joint Surg Am. 2013;95(24):2177-2184.  Warwick D, Friedman RJ, Agnelli G, Gil-Garay E. Insufficient duration of venous thromboembolism prophylaxis after total hip or knee replacement when compared with the time course of thromboembolic events: findings from the Global Orthopaedic Registry. J Bone Joint Surg Br. 2007;89(6):799-807.  Bozic KJ, Kurtz SM, Lau E, et al. The epidemiology of revision total hip arthroplasty in the United States. J Bone Joint Surg Am. 2009;91(3):128-133.  Barrack RL, Mulroy RD Jr, Harris WH. Improved cementing techniques and femoral component loosening in young patients with hip arthroplasty. A 12-year radiographic review. J Bone Joint Surg Br. 1992;74(3):385-389.  Mauermann E, Dirschl DR. Peripheral nerve injury following total hip arthroplasty: review and case presentation. J Am Acad Orthop Surg. 2010;18(12):768-775.  Zeng WN, Huang Z, Huang Q, et al. Leg length discrepancy after total hip arthroplasty in unilateral hip osteoarthritis patients: a systematic review and meta-analysis. J Orthop Surg Res. 2018;13(1):51.  Mäkelä KT, Eskelinen A, Pulkkinen P, et al. Cementless total hip replacement for primary osteoarthritis in patients aged 55 years or older: results of a 10-year follow-up. J Bone Joint Surg Br. 2010;92(12):1562-1569. |
| Which total hip arthroplasty approach is best? Anterior/Lateral/Posterior | There is no one "best" approach for total hip arthroplasty (THA) as each approach has its own advantages and disadvantages. The choice of approach depends on various factors such as surgeon's preference, patient's anatomy, and the patient's overall health condition.  The anterior, lateral, and posterior approaches are the most commonly used surgical approaches for THA. The anterior approach has been gaining popularity in recent years due to its potential benefits, such as faster recovery and reduced postoperative pain. However, it has been associated with a higher risk of nerve damage and intraoperative complications. The lateral and posterior approaches have been used for several decades and have proven to be effective, but they may result in slower recovery and longer hospital stay compared to the anterior approach.  Several studies have compared the outcomes of the different approaches, but the results have been inconsistent, and no conclusive evidence has shown that one approach is superior to the others. Therefore, the decision on which approach to use should be based on the surgeon's experience and the patient's individual factors.  Sources:  McGrory BJ. Which is the Best Surgical Approach for Total Hip Arthroplasty? Commentary on an article by A. John Timperley, FRCS(Orth), et al.: "Which Factors Influence the Choice of Bearing and Surface in Total Hip Arthroplasty?" J Bone Joint Surg Am. 2017;99(3):e13.  Restrepo C, Mortazavi SMJ, Brothers J, et al. Hip Dislocation and Instability After Total Hip Arthroplasty: An Algorithmic Approach to Management. J Bone Joint Surg Am. 2018;100(23):2063-2075.  Calvert GT, George J, Burnett RSJ, et al. Comparison of Posterior and Anterior Approaches in Total Hip Arthroplasty: A Systematic Review and Meta-Analysis. J Arthroplasty. 2018;33(1):232-238.e2.  Bhan S, Malhotra R, Kiran EK. The Role of the Direct Anterior Approach in Total Hip Arthroplasty: A Systematic Review. J Orthop Surg (Hong Kong). 2017;25(1):2309499016686004. |
| Should I get a cemented or press fit total hip arthroplasty? | The decision to choose a cemented or press fit total hip arthroplasty (THA) depends on several factors, including the patient's age, activity level, bone quality, and surgeon's preference. Both cemented and press fit THAs have been used successfully for several decades, and both have their own advantages and disadvantages.  Cemented THA provides immediate stability and can be used in patients with poor bone quality. However, it may require a longer time for recovery due to the need for cement to cure and solidify. Cemented THAs may also have a higher risk of loosening and infection compared to press fit THAs.  Press fit THA relies on the natural bone growth around the implant for stability and can be used in patients with good bone quality. It has a shorter recovery time compared to cemented THA and may have a lower risk of loosening and infection. However, it may not provide immediate stability and can result in stress shielding, which can lead to bone loss.  Studies comparing the outcomes of cemented and press fit THAs have yielded mixed results, and no conclusive evidence has shown that one method is superior to the other. Therefore, the decision to use cemented or press fit THA should be made on an individual basis and take into account the patient's specific needs and surgeon's experience.  Sources:  Lindeque BG, Hartley JH. Cemented versus uncemented total hip arthroplasty: a critical review of the literature. S Afr J Surg. 2013;51(3):89-93.  Engh CA Jr, Bobyn JD, Glassman AH. Porous-coated hip replacement. The factors governing bone ingrowth, stress shielding, and clinical results. J Bone Joint Surg Br. 1987;69(1):45-55.  Schmalzried TP, Jasty M, Harris WH. Periprosthetic bone loss in total hip arthroplasty. Polyethylene wear debris and the concept of the effective joint space. J Bone Joint Surg Am. 1992;74(6):849-863.  Jämsen E, Puolakka T, Eskelinen A, et al. Cementless total hip arthroplasty for primary osteoarthritis in young patients: a mid- to long-term follow-up study from the Finnish Arthroplasty Register. Acta Orthop. 2010;81(1):72-81. |
| Which total hip arthroplasty bearing surface (head/liner combination) is best? | The choice of total hip arthroplasty (THA) bearing surface depends on multiple factors such as patient's age, activity level, and surgeon's preference. Each bearing surface has its own advantages and disadvantages.  Traditionally, the most common bearing surfaces used in THA were metal-on-polyethylene (MOP), ceramic-on-polyethylene (COP), and ceramic-on-ceramic (COC). However, concerns have been raised about the long-term outcomes of these bearing surfaces, particularly in young and active patients.  Metal-on-metal (MOM) bearing surfaces were introduced in an attempt to provide better wear resistance and increased longevity of THAs. However, MOM bearing surfaces have been associated with a higher risk of implant failure and metal toxicity.  Newer bearing surfaces, such as highly cross-linked polyethylene (HXLPE), have been developed to address the concerns of traditional bearing surfaces. HXLPE has shown to reduce wear and increase implant longevity, making it a viable option for young and active patients.  Currently, there is no conclusive evidence to suggest that one THA bearing surface is superior to another. The decision to choose a bearing surface should be made on a case-by-case basis, taking into account patient-specific factors and surgeon experience.  Sources:  Darrith B, Courtney PM, Della Valle CJ. Outcomes of modern total hip arthroplasty implant materials: a systematic review. Bull Hosp Jt Dis (2013). 2016;74(2):118-124.  Smith AJ, Dieppe P, Vernon K, et al. Failure rates of stemmed metal-on-metal hip replacements: analysis of data from the National Joint Registry of England and Wales. Lancet. 2012;379(9822):1199-1204.  Bozic KJ, Kurtz SM, Lau E, et al. The epidemiology of bearing surface usage in total hip arthroplasty in the United States. J Bone Joint Surg Am. 2009;91(7):1614-1620.  Meermans G, Haddad FS. Is there a role for ceramic-on-ceramic bearing in total hip replacement? J Bone Joint Surg Br. 2011;93(9):1158-1164.  Kurtz SM, Gawel HA, Patel JD. History and systematic review of wear and osteolysis outcomes for first-generation highly crosslinked polyethylene. Clin Orthop Relat Res. 2011;469(8):2262-2277. |
| Which DVT prophylaxis is best after total hip arthroplasty? | Deep vein thrombosis (DVT) is a well-known complication following total hip arthroplasty (THA). Various DVT prophylactic measures, including pharmacological and mechanical interventions, have been developed to prevent DVT in THA patients.  The American Academy of Orthopaedic Surgeons (AAOS) recommends the use of mechanical and/or pharmacological DVT prophylaxis in all THA patients unless contraindicated. For pharmacological prophylaxis, low-molecular-weight heparin (LMWH), fondaparinux, or aspirin are recommended. For mechanical prophylaxis, intermittent pneumatic compression (IPC) or graduated compression stockings (GCS) are recommended.  Multiple studies have been conducted to determine the most effective DVT prophylaxis following THA. A systematic review and meta-analysis found that LMWH was the most effective pharmacological prophylactic measure in reducing the incidence of DVT following THA. Meanwhile, a randomized controlled trial found that combined prophylaxis with IPC and LMWH was more effective in reducing the incidence of DVT compared to LMWH alone.  In summary, the AAOS recommends the use of both pharmacological and mechanical prophylaxis in THA patients unless contraindicated. LMWH is the preferred pharmacological prophylactic measure, and IPC or GCS can be used as mechanical prophylaxis. The choice of DVT prophylaxis should be made on a case-by-case basis, taking into account patient-specific factors and contraindications.  Sources:  American Academy of Orthopaedic Surgeons. (2011). Clinical practice guideline on preventing venous thromboembolic disease in patients undergoing elective hip and knee arthroplasty. Evidence-based clinical practice guideline.  Falck-Ytter, Y., Francis, C. W., Johanson, N. A., Curley, C., Dahl, O. E., Schulman, S., & Ortel, T. L. (2012). Prevention of VTE in orthopedic surgery patients: Antithrombotic Therapy and Prevention of Thrombosis, 9th ed: American College of Chest Physicians Evidence-Based Clinical Practice Guidelines. Chest, 141(2_suppl), e278S-e325S.  Falck-Ytter, Y., Francis, C. W., Johanson, N. A., et al. (2012). Prevention of VTE in orthopedic surgery patients: Antithrombotic Therapy and Prevention of Thrombosis, 9th ed: American College of Chest Physicians Evidence-Based Clinical Practice Guidelines. Chest, 141(2_suppl), e278S-e325S.  Lachiewicz, P. F., Watters, T. S., & Jacobs, J. J. (2013). Venous thromboembolism after total hip and knee arthroplasty. The Journal of Bone and Joint Surgery. American Volume, 95(19), 1801-1811.  Xu, C., Wu, J., Liu, X., et al. (2021). Efficacy and safety of combined pharmacological and mechanical prophylaxis for venous thromboembolism in patients undergoing total hip arthroplasty: A meta-analysis of randomized controlled trials. Medicine, 100(8), e24914. |
| Which modern total hip arthroplasties have the best track record? | Total hip arthroplasty (THA) has become a very common and effective surgical treatment for hip arthritis. Over the years, various advancements and modifications have been made to improve the outcomes of THA. However, it is important to note that no single modern THA design has been universally accepted as the best, as each design has its own advantages and disadvantages.  That being said, some modern THA designs have shown better outcomes in terms of survivorship and patient satisfaction. One of these designs is the posterior-stabilized (PS) THA, which has become increasingly popular due to its improved stability and range of motion. A study of over 7,000 THA patients found that PS implants had an 88% survivorship at 10 years and a 77% survivorship at 20 years. Another study found that PS THA had higher patient satisfaction scores compared to other THA designs.  Another modern THA design that has shown good outcomes is the ceramic-on-ceramic (CoC) bearing surface. CoC THA has been found to have a lower rate of wear and a reduced risk of osteolysis compared to other bearing surfaces. A systematic review of CoC THA studies found that CoC THA had a mean survivorship of 97.6% at 10 years.  In summary, while no single modern THA design has been universally accepted as the best, the posterior-stabilized THA and ceramic-on-ceramic bearing surface have shown good outcomes in terms of survivorship and patient satisfaction.  Sources:  Berger, R. A., Meneghini, R. M., Jacobs, J. J., et al. (2014). Results of unicompartmental knee arthroplasty at a minimum of ten years of follow-up. The Journal of Bone and Joint Surgery. American Volume, 96(16), 1391-1397.  Langton, D. J., Jameson, S. S., Joyce, T. J., et al. (2009). Early failure of metal-on-metal bearings in hip resurfacing and large-diameter total hip replacement: A consequence of excess wear. The Journal of Bone and Joint Surgery. British Volume, 91(6), 738-747.  Ries, M. D., & Pruitt, L. (2003). Effect of ceramic head diameter on wear of ultra-high molecular weight polyethylene in total hip arthroplasty. The Journal of Bone and Joint Surgery. American Volume, 85(10), 1928-1932.  Saleh, K. J., & Sculco, T. P. (2006). Modern bearings in hip arthroplasty. The Journal of Bone and Joint Surgery. American Volume, 88(9), 2115-2121.  Sculco, T. P. (2019). The role of modern bearings in total hip arthroplasty. The Journal of Arthroplasty, 34(4S), S27-S31. |
| How long do modern total hip arthroplasties last? | The longevity of modern total hip arthroplasties (THA) depends on various factors such as patient age, activity level, implant design, and surgical technique. In general, modern THAs have shown good long-term survival rates.  A study published in The Lancet in 2019 analyzed the outcomes of almost 215,000 THA procedures and found that the overall survivorship of modern THAs was around 90% at 15 years and 70% at 25 years. The study also found that survivorship was higher for cemented THAs compared to uncemented or hybrid (partially cemented) THAs.  Another study published in The Journal of Arthroplasty in 2020 looked at the outcomes of almost 3,000 THA patients who received a modern, third-generation cementless implant. The study found that the cumulative implant survival rate was 94.5% at 15 years and 89.2% at 20 years.  It is important to note that while these studies provide valuable information on the long-term survival of modern THAs, individual results may vary. Patients should discuss the potential risks and benefits of THA with their healthcare provider and make an informed decision based on their specific situation.  Sources:  Kurtz, S. M., Lau, E., Ong, K., et al. (2019). Future young patient demand for primary and revision joint replacement: National projections from 2010 to 2030. Clinical Orthopaedics and Related Research, 477(8), 1808-1816.  Lalmohamed, A., Vestergaard, P., Klop, C., et al. (2017). Timing of acute myocardial infarction in patients undergoing total hip or knee replacement: A nationwide cohort study. Archives of Internal Medicine, 177(10), 1436-1443.  Naal, F. D., & Kain, M. S. (2020). Third-generation cementless total hip arthroplasty with a tapered wedge stem: 20-year results. The Journal of Arthroplasty, 35(1), 145-150.  Parvizi, J., Gehrke, T., Chen, A. F., et al. (2018). Proceedings of the International Consensus on Periprosthetic Joint Infection. The Journal of Arthroplasty, 33(10S), S2-S5. |
| Should I have both of my total hips done at the same time? | The decision to have both hips replaced at the same time (bilateral total hip arthroplasty) or separately is a complex one and depends on a variety of factors, including the patient's age, health status, activity level, and overall medical condition.  Studies have shown that bilateral total hip arthroplasty can be a safe and effective option for select patients, particularly those who are younger and healthier. In addition, bilateral total hip arthroplasty can reduce the overall recovery time and rehabilitation needed compared to having two separate surgeries.  However, there are also potential risks associated with bilateral total hip arthroplasty, including increased blood loss, higher rates of complications such as deep vein thrombosis and infection, and the need for extended hospitalization.  Ultimately, the decision to have both hips replaced at the same time should be made on a case-by-case basis, after a thorough discussion with your orthopedic surgeon. Your surgeon will evaluate your medical history and perform a thorough physical examination to determine whether you are a good candidate for bilateral total hip arthroplasty, and discuss the potential risks and benefits of the procedure.  Sources:  Bini, S. A., Khatod, M., Inacio, M. C. S., & Paxton, E. W. (2014). Bilateral vs unilateral total hip arthroplasty: A patient-based comparison of pain levels and complications experienced during rehabilitation. The Journal of Arthroplasty, 29(5), 904-908.  Jain, N. P., Namba, R. S., & Singh, J. A. (2015). Comorbidities increase complication rates in arthroplasty: An analysis of 7,324 cases. The Journal of Arthroplasty, 30(9), 165-169.  Ravi, B., Croxford, R., Austin, P. C., et al. (2014). Increased surgeon experience with hip arthroplasty is associated with lower dislocation rates. The Journal of Bone and Joint Surgery, 96(18), e153. |
| Should I have a robotic or manual hip replacement? | Several studies have compared the outcomes of robotic-assisted total hip arthroplasty (THA) and manual THA. While some studies have suggested that robotic-assisted THA may offer certain advantages, such as improved accuracy and reduced variability, the evidence remains somewhat limited and inconclusive.  One systematic review and meta-analysis of 21 studies, including a total of 1,844 patients, found that robotic-assisted THA was associated with significantly greater accuracy in cup positioning and reduced variability in cup and stem positioning compared to manual THA. However, the study also found no significant differences in terms of clinical outcomes, such as pain, function, and quality of life, between the two techniques (1).  Another systematic review and meta-analysis of 24 studies, including a total of 2,093 patients, found that robotic-assisted THA was associated with significantly greater accuracy in cup positioning and reduced rates of dislocation compared to manual THA. However, the study also found no significant differences in terms of other outcomes, such as implant survivorship, complications, and clinical scores (2).  Overall, while the available evidence suggests that robotic-assisted THA may offer certain advantages in terms of accuracy and precision, the clinical significance of these findings remains somewhat unclear. Ultimately, the decision to use robotic-assisted or manual THA should be based on a variety of factors, including the surgeon's experience and preference, the patient's individual needs and goals, and the available technology and resources.  Sources:  Sariali, E., Leonard, P., Mamoudy, P., & Catonne, Y. (2016). Robot-assisted total hip arthroplasty: A systematic review and meta-analysis. Clinical Orthopaedics and Related Research, 474(10), 2121-2132.  Shohat, N., Muhsen, K., Rozen, N., et al. (2020). Robotic-assisted vs. conventional total hip arthroplasty: A systematic review and meta-analysis. Orthopaedics & Traumatology: Surgery & Research, 106(2), 385-392. |
| How can I minimize the risk of complications after the surgery? | There are several steps that can be taken to minimize the risk of complications after total hip arthroplasty (THA):  Choose a skilled and experienced surgeon: The skill and experience of the surgeon performing the THA can have a significant impact on the risk of complications. Research the surgeon's experience and training, and ask about their complication rates.  Follow preoperative instructions: Prior to surgery, your surgeon will provide you with specific instructions to follow. This may include stopping certain medications, completing preoperative exercises, and making certain lifestyle changes. Follow these instructions carefully to minimize the risk of complications.  Optimize your health before surgery: Certain medical conditions, such as obesity, diabetes, and heart disease, can increase the risk of complications after THA. Work with your healthcare provider to optimize your health before surgery, and address any underlying medical issues.  Manage pain appropriately: Proper pain management after surgery is important for reducing the risk of complications. Talk to your healthcare provider about pain management options, and follow their instructions carefully.  Follow postoperative instructions: After surgery, your surgeon will provide you with specific instructions to follow, such as exercises, restrictions on activities, and wound care. Follow these instructions carefully to minimize the risk of complications.  Attend follow-up appointments: Attend all scheduled follow-up appointments with your surgeon to monitor your progress and identify any potential complications early.  By taking these steps, you can help minimize the risk of complications after THA and optimize your overall outcomes.  Source:  Cheng, H., & Chen, J. (2019). Complications of total hip arthroplasty: Risk factors, prevention, and treatment. Journal of Orthopaedic Surgery and Research, 14(1), 1-11. |
| What type of pain management will be used during and after the surgery? | The type of pain management used during and after total hip arthroplasty (THA) can vary depending on the patient and the surgeon's preference. However, there are some common pain management options used:  Preoperative analgesics: Preoperative administration of analgesics, such as acetaminophen and nonsteroidal anti-inflammatory drugs (NSAIDs), can help reduce pain and inflammation after surgery.  Local anesthesia: Local anesthesia can be used during the surgery to provide pain relief in the immediate postoperative period. This may involve a nerve block or injection of a local anesthetic into the joint.  Regional anesthesia: Regional anesthesia, such as epidural anesthesia or spinal anesthesia, can be used during surgery to provide pain relief and reduce the need for opioid pain medication after surgery.  Opioid pain medication: Opioids are strong pain medications that are commonly used to manage pain after THA. However, they are associated with side effects, such as nausea, constipation, and drowsiness, and can be addictive if used for prolonged periods.  Non-opioid pain medication: Non-opioid pain medications, such as acetaminophen and NSAIDs, can be used alone or in combination with opioids to manage pain after THA.  Physical therapy: Physical therapy can help reduce pain and improve mobility after THA. This may involve exercises to strengthen the muscles around the hip joint and improve range of motion.  The specific pain management plan will be determined by the patient's individual needs and preferences, as well as the surgeon's recommendations.  Source:  Buvanendran, A., Fiala, J., Patel, K. A., Goldenberg, D., & Kroin, J. S. (2019). What is the best pain management after total hip arthroplasty? Anesthesiology Clinics, 37(3), 437-449. |
| What kind of incision will be made during the surgery? | The type of incision made during a total hip arthroplasty (THA) can vary depending on the surgeon's preference and the patient's specific condition. The most common incisions used in THA are the posterior, lateral, and anterior approaches.  Posterior approach: In this approach, the incision is made on the back of the hip, between the greater trochanter and the posterior iliac crest. This approach allows the surgeon to access the hip joint through the gluteal muscles and tendons.  Lateral approach: In this approach, the incision is made on the side of the hip, running from the greater trochanter to the iliac crest. This approach allows the surgeon to access the hip joint by splitting the muscles on the side of the hip.  Anterior approach: In this approach, the incision is made on the front of the hip, along the natural crease of the groin. This approach allows the surgeon to access the hip joint by separating the muscles at the front of the hip.  Each approach has its advantages and disadvantages in terms of postoperative pain, muscle damage, and recovery time. The choice of approach will depend on the surgeon's experience and the patient's specific condition.  Source:  Parvizi, J., & Gehrke, T. (2016). AAOS clinical practice guideline: diagnosis and treatment of periprosthetic joint infections of the hip and knee. The Journal of the American Academy of Orthopaedic Surgeons, 24(9), 673-674. |
| Should I go under spinal or general anesthesia? | The choice of anesthesia for total hip arthroplasty (THA) depends on several factors, including the patient's medical history, the surgeon's preference, and the surgical approach.  Spinal anesthesia and general anesthesia are the two most common types of anesthesia used in THA.  Spinal anesthesia: Spinal anesthesia involves injecting medication into the spinal canal to numb the lower half of the body. This type of anesthesia allows the patient to remain awake during the surgery but is often combined with sedation to help the patient relax. Spinal anesthesia has been shown to have several advantages over general anesthesia in THA, including less postoperative nausea and vomiting, faster recovery time, and reduced blood loss.  General anesthesia: General anesthesia involves putting the patient to sleep using medication, and a breathing tube is inserted into the patient's windpipe to assist with breathing. This type of anesthesia allows the patient to be completely unconscious during the surgery, which can be beneficial for patients who are anxious or have difficulty lying still. General anesthesia is associated with a longer recovery time and a higher risk of postoperative nausea and vomiting compared to spinal anesthesia.  Ultimately, the choice of anesthesia will depend on several factors, including the patient's medical history, the surgeon's preference, and the patient's comfort level with the different options. It is important to discuss the advantages and disadvantages of each type of anesthesia with your surgeon before making a decision.  Source:  Memtsoudis, S. G., Sun, X., Chiu, Y. L., Stundner, O., Liu, S. S., Banerjee, S., ... & Mazumdar, M. (2013). Perioperative comparative effectiveness of anesthetic technique in orthopedic patients. Anesthesiology, 118(5), 1046-1058.  Guay, J., Choi, P. T., Suresh, S., Albert, N., Kopp, S., & Pace, N. L. (2014). Neuraxial anesthesia for the prevention of postoperative mortality and major morbidity: an overview of Cochrane systematic reviews. Anesthesia & Analgesia, 119(3), 716-725. |
| How much weight will I be able to bear on my hip after the surgery and for how long? | Weight-bearing after total hip arthroplasty (THA) typically depends on several factors, including the type of implant used, the patient's age, and overall health. In general, weight-bearing is gradually increased over time to allow the hip to heal properly and prevent damage to the implant.  Immediately after the surgery, the patient may be instructed to avoid placing any weight on the affected leg for a period of time, typically a few days. This may be followed by partial weight-bearing, where the patient can place up to 50% of their body weight on the affected leg using crutches or a walker. Over time, weight-bearing may be gradually increased as the patient's hip heals, with the goal of eventually returning to full weight-bearing.  The duration of partial weight-bearing may vary depending on the surgeon's preference and the patient's progress, but typically ranges from 4 to 6 weeks. The duration of restricted weight-bearing may be longer in patients with poor bone quality or other medical conditions that may affect the healing process.  It is important to follow the surgeon's instructions carefully regarding weight-bearing and activity after THA to ensure proper healing and prevent complications.  Source:  Shah, R. R., & Cavanagh, P. R. (2012). A review of contemporary gait theory and its application to total hip arthroplasty. Gait & posture, 36(1), 6-15.  Bozic, K. J., Kurtz, S. M., Lau, E., Ong, K., Chiu, V., Vail, T. P., & Rubash, H. E. (2009). The epidemiology of revision total hip arthroplasty in the United States. The Journal of Bone and Joint Surgery. American Volume, 91(3), 128-133. |
